# Elevated AST/ALT ratio is associated with all‐cause mortality and cancer incident

**DOI:** 10.1002/jcla.24356

**Published:** 2022-03-22

**Authors:** Wangyang Chen, Weibo Wang, Lingling Zhou, Jun Zhou, Lianping He, Jiayi Li, Xinyue Xu, Jixi Wang, Liangyou Wang

**Affiliations:** ^1^ School of Medicine Taizhou University Taizhou Zhejiang Province China; ^2^ Department of General Practice BaiYun Community Health Service Center Taizhou Zhejiang Province China; ^3^ Department of Anatomy College of Medicine Jiaxing University Jiaxing Zhejiang Province China; ^4^ Department of Non‐Communicable Chronic Disease Control and Prevention Taizhou Center for Disease Control and Prevention Taizhou Zhejiang Province China

**Keywords:** aminotransferase, AST/ALT ratio, cancer, chronic disease, mortality

## Abstract

**Background:**

The aspartate transaminase (AST)‐to‐alanine aminotransferase (ALT) ratio, which is used to measure liver injury, has been found to be associated with some chronic diseases and mortality. However, its relevance to cancer incidence resulting from population‐based prospective studies has rarely been reported. In this study, we investigated the correlation of the AST/ALT ratio as a possible predictor of mortality and cancer incidence.

**Methods:**

A total of 9,946 participants fulfilled the inclusion criteria for a basic public health service project of the Health Checkup Program conducted by the BaiYun Community Health Service Center, Taizhou. Deceased participants and cancer incident cases were from The Taizhou Chronic Disease Information Management System. Odds ratios (ORs) and interval of quartile range (IQR) computed by logistic regression analysis and cumulative incidence rate were calculated by the Kaplan–Meier survival method and compared with log‐rank test statistics.

**Results:**

Serum ALT and AST levels were both increased in patients with chronic diseases, but the ratio of AST/ALT was generally decreased. The cancer incident cases (488 new cases) had a greater baseline ratio (median =1.23, IQR: 0.96–1.54) than noncancer cases (median =1.15, IQR: 0.91–1.44). Compared to the first quartile of the AST/ALT ratio, the population in the top quartile had a higher cumulative cancer incidence rate (7.54% vs. 4.44%) during follow‐up period. Furthermore, an elevated AST/ALT ratio increased the risk of all‐cause mortality.

**Conclusions:**

The ratio of AST/ALT is a potential biomarker to assess healthy conditions and long‐term mortality. Especially for cancer, the AST/ALT ratio not only increases at baseline but also predicts the future development of cancer. The clinical value and potential mechanism deserve further research.

## Graphical abstract

At follow‐up, 494 individuals were newly diagnosed with cancer and the cumulative incidence rate was 5.63%. Four groups were divided based on the quartile of the AST/ALT ratio. The incidence rates of 4.44% was in the group at the first quartile of the AST/ALT ratio, 4.93% for the second quartile, 5.62% for the third quartile, and 7.54% for the fourth quartile.

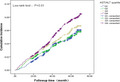

## 1 INTRODUCTION

Noncommunicable chronic diseases, including hypertension, type2 diabetes mellitus (T2DM), cancer, stroke, fatty liver, etc., are threatening to human health and contribute to the death of the population. Considerable evidence has demonstrated that smoking, drinking, physical inactivity, and obesity are common risk factors for the abovementioned chronic diseases.^1^ Oxidative stress and systematic inflammation are two plausible mechanisms of chronic diseases.^2^, ^3^ Therefore, many oxidative stress and systematic inflammation markers, such as serum malondialdehyde (MDA), superoxide dismutase (SOD), albumin (ALB), and neutrophil‐to‐lymphocyte ratio (NLR), have been identified and used for screening or evaluating the health condition of chronic diseases in the population.^4^, ^5^, ^6^, ^7^ Most of them have performed imperfectly in clinical practice. Thus, more effective biomarkers to assess health conditions and predict disease development are needed.

Aminotransaminases, including aspartate transaminase (AST) and alanine aminotransferase (ALT), which are well‐known blood‐based circulating biomarkers, have always been used to reflect liver injury. However, it has also been found that increased aminotransferases are associated with human diseases and systemic regulation of metabolic functions.^8^ The concept of aspartate transaminase/alanine transaminase (AST/ALT), also termed the De Ritis ratio, was first proposed for the study of hepatitis etiology^9^ and commonly used to differentiate varying causes of liver disease such as fatty liver. Currently, the AST/ALT ratio is also used as an effective biomarker for nonliver diseases, such as cardiovascular disease,^10^ various cancers,^11^, ^12^, ^13^ and T2DM.^14^ Initially, a high AST/ALT ratio was reported to predict poor prognosis in nonmetastatic renal cell carcinoma.^15^ Since then, more retrospective studies have verified the association of the AST/ALT ratio with cancer prognosis.

Previous reports focused more on the association of the AST/ALT ratio with mortality or cancer survival but few studies evaluate the predictive value of the AST/ALT ratio for cancer onset and its association with chronic diseases. To detect the long‐term effect on health, the aim of this study was to evaluate the AST/ALT ratio as a potential predictive biomarker for all‐cause mortality and cancer incidence.

## 2 MATERIALS AND METHODS

### 2.1 Study population

The study population consisted of 10,657 sampled persons aged 40 years and older residing in the BaiYun community who participated in the basic public health service project of the Health Checkup Program from May 2015 to November 2019. The survey consisted of cross‐sectional interviews, anthropometric measures, and laboratory tests performed at the BaiYun Community Health Service Center in Taizhou. For analyses of aminotransferases, we excluded persons who lacked data on ALT or AST (*n* = 711), which resulted in an analysis sample of 9,946. This study protocol and procedure were approved according to the ethical standards of the Declaration of Helsinki, and all participants provided informed consent.

### 2.2 Laboratory tests

Fast anticoagulant venous blood sampling was drawn, and the concentrations of serum AST (normality range: 0–45 U/L) and ALT (normality range: 0–50 U/L) were determined using the enzymatic rate method according to the International Federation of Clinical Chemistry recommendation (BS‐820 M, Mindray System‐Mindray Diagnostics). The AST‐to‐ALT ratio was calculated as the AST divided by ALT. Fasting blood glucose levels were detected using the spectrophotometry (glucose oxidase) method and Glucose (GOD) Kit diagnostic reagent (Mindray Co, Ltd).

Exposure information was collected as a covariate for this study: demographic information (age, sex, and BMI) and lifestyle information (smoking status, alcohol consumption, and exercise frequency). BMI was categorized into four groups (<18.5, 18.5–24.9, 25.0–29.9, and >30 kg/m^2^). Smoking status was divided into three groups (never smoker, previous smoker, and current smoker). Alcohol consumption was categorized into four groups (never drink, <1 time/month, <3 times/week, and 3 times/week‐everyday drinking in the last 2 years). Exercise frequency was divided into four groups (inactive: seldom exercise, moderately inactive: 1–2 times per week, moderately active: 3–4 times per week, and active: ≥5 times per week).

### 2.3 Diagnostic criteria and definitions

Chronic diseases in this study included hypertension, T2DM, cancer, stroke, and fatty liver disease. The medical history of individuals was provided by self‐reported answers to a questionnaire.

Hypertension was defined as being diagnosed with hypertension or taking antihypertensive drugs for more than 3 months, and potential hypertension was defined when diastolic pressure was higher than 90 mmHg or systolic pressure was higher than 140 mmHg; T2DM was defined as a previous diagnosis of diabetes mellitus by self‐report or the use of any hypoglycemic drugs. Hyperglycemia referred to fasting blood glucose levels >6.1 mmol/L in this survey and never determined by clinicians before. Fatty liver was ascertained among participants by abdominal ultrasound. The diagnostic criteria were based on the presence of hepatorenal echo contrast, liver parenchymal brightness, deep attenuation, and vascular blurring.^16^ Cancer was defined as a previous diagnosis of cancer by self‐report or recorded in the cancer registry, and the diagnosis date was in head of enrollment in this study. Stroke was defined according to the presenting symptoms, such as awakening with or experiencing the abrupt onset of focal neurological deficits and noncontrast head computed tomography imaging indicating hemorrhage or ischemic change, as recorded in the medical records.

### 2.4 Information of death and incident cancer

Individual data included in this study were linked to The Taizhou Chronic Disease Information Management System and The Cause of Death Registration and Reporting Information System from Taizhou Center for Disease Control and Prevention. The death information included direct and indirect causes of death and the death date. The new cancer cases were defined as ICD‐10 coding from C00 to C97, and benign brain tumors were diagnosed in participants after they joined the present study. Death and cancer incident data were analyzed until December 31, 2020.

### 2.5 Statistical analysis

Data are shown as the mean and standard deviation when normally distributed or expressed as the median with interquartile range. Categorical data are expressed as numbers and percentages. Levels of the AST/ALT ratio among different groups were compared by the nonparameter Kruskal–Wallis test and Chi‐squared tests when used for categorical variables. The correlation coefficients (*r*) between two variables were calculated by the Spearman correlation analysis. The association between mortality and the AST/ALT ratio was evaluated by multivariable logistic regression analysis after adjusting for age, sex, smoking, drinking, physical activity, and BMI. The cancer incidence rate between each quartile of AST/ALT ratio was calculated by the Kaplan–Meier survival method and compared with log‐rank statistics. Subgroup analysis was conducted by logistic analysis, and the reference of each variable was defined as the lowest level of age group, BMI level, and never drinking, smoking, or physical activity. The data were analyzed using GraphPad Prism software version 6.0 (San Diego) and IBM SPSS 22.0 (IBM Corp.). *p* values were two‐sided, and a *p* value of < 0.05 was considered to indicate statistical significance.

## 3 RESULTS

### 3.1 Characteristics of participants

Of the 9,765 participants enrolled in this study, four groups were assembled by quartile according to the AST/ALT ratio. The AST/ALT ratio was significantly different among the participants grouped by age, BMI, physical activity, smoking status, and drinking behavior. The mean age increased with the AST/ALT ratio, and the mean ages from quartile 1 to quartile 4 were 59.29, 62.68, 64.44, and 67.83, respectively. Conversely, the higher the AST/ALT ratio, the lower the mean BMI was (BMI 23.53 ± 3.27 for quartile 4 vs. 26.21 ± 3.35 quartile 1). In the chronic diseases, more cases of each type of disease were preponderant in the group with a low AST/ALT ratio. There were 335 cancer cases at baseline and 494 incident cases during the follow‐up period. The prevalence of fatty liver was 22.66% among all surveyed populations. Because of hepatobiliary diseases’ influence on AST and ALT level, we performed sensitivity analysis to show the distribution of AST/ALT ratio in patients with fatty liver was attenuated to null after excluding patients with cholecystitis, gallstone, and liver cirrhosis (Table S1). Information on behaviors, such as smoking, drinking, and physical activity, in each group is shown in Table 1.

**TABLE 1:** Characteristics of participants in the study

	AST/ALT ratio				*χ* ^2^/F	*p*
	Quartile 1 (%)	Quartile 2 (%)	Quartile 3 (%)	Quartile 4 (%)		
No.	2,499	2,407	2,577	2,463		
Gender					3.969	0.265
Male	1,094 (43.78)	1,070 (44.45)	1,087 (42.18)	1,101 (44.7)		
Female	1,405 (56.22)	1,337 (55.55)	1,490 (57.82)	1,362 (55.3)		
Age	59.29 ± 8.91	62.68 ± 8.81	64.44 ± 9.58	67.82 ± 10.62	347.6	<0.001
40	336 (13.45)	154 (6.4)	173 (6.71)	129 (5.24)	1068.32	<0.001
50	955 (38.22)	689 (28.62)	563 (21.85)	356 (14.45)		
60	884 (35.37)	1,037 (43.08)	1,066 (41.37)	886 (35.97)		
70	281 (11.24)	446 (18.53)	612 (23.75)	715 (29.03)		
80	43 (1.72)	81 (3.37)	163 (6.33)	377 (15.31)		
BMI	26.21 ± 3.35	25.48 ± 3.23	24.60 ± 3.10	23.53 ± 3.27	313.505	<0.001
<18.5	10 (0.4)	20 (0.83)	36 (1.4)	96 (3.91)		
18.5	918 (36.81)	1,051 (43.81)	1,452 (56.48)	1,648 (67.07)	763.943	<0.001
25	1,264 (50.68)	1,144 (47.69)	954 (37.11)	629 (25.6)		
>30	302 (12.11)	184 (7.67)	129 (5.02)	84 (3.42)		
Physical activity					45.904	<0.001
Inactive	1,236 (49.54)	1,199 (49.96)	1,301 (50.66)	1,394 (56.87)		
Moderately inactive	423 (16.95)	392 (16.33)	386 (15.03)	334 (13.63)		
Moderately active	176 (7.05)	135 (5.63)	176 (6.85)	122 (4.98)		
Active	660 (26.45)	674 (28.08)	705 (27.45)	601 (24.52)		
Smoking					14.172	0.028
Current	440 (17.61)	386 (16.04)	370 (14.36)	382 (15.51)		
Previous	177 (7.09)	183 (7.6)	169 (6.56)	161 (6.54)		
Never	1,881 (75.3)	1,838 (76.36)	2,037 (79.08)	1,920 (77.95)		
Drinking					66.998	<0.001
Never	1,768 (70.83)	1,681 (69.9)	1,845 (71.62)	1,769 (71.85)		
<1 time/month	415 (16.63)	369 (15.34)	318 (12.34)	264 (10.72)		
>3 times/week	138 (5.53)	178 (7.40)	174 (6.75)	188 (7.64)		
Everyday	175 (7.01)	177 (7.36)	239 (9.28)	241 (9.79)		
Hypertension						
No	698 (27.93)	714 (29.66)	845 (32.79)	886 (35.97)	97.307	<0.001
Yes	1,398 (55.94)	1,265 (52.56)	1,294 (50.21)	1,052 (42.71)		
Potential	403 (16.13)	428 (17.78)	438 (17.00)	525 (21.32)		
Diabetes					385.109	<0.001
No	1,404 (56.18)	1,511 (62.78)	1,837 (71.28)	1,967 (79.86)		
Yes	587 (23.49)	446 (18.53)	326 (12.65)	209 (8.49)		
Potential	508 (20.33)	450 (18.70)	414 (16.07)	287 (11.65)		
Cancer					25.825	<0.001
No	2,314 (92.6)	2,206 (91.65)	2,372 (92.05)	2,225 (90.34)		
Incident	99 (3.96)	104 (4.32)	129 (5.01)	162 (6.58)		
Prevalent	86 (3.44)	97 (4.03)	76 (2.95)	76 (3.09)		
Stroke					6.716	0.082
No	2,440 (97.83)	2,361 (98.29)	2,511 (97.51)	2,395 (97.24)		
Yes	54 (2.17)	41 (1.71)	64 (2.49)	68 (2.76)		
Fatty liver					1097.981	<0.001
No	1,417 (56.82)	1,761 (73.31)	2,225 (86.41)	2,294 (93.14)		
Yes	1,077 (43.18)	641 (26.69)	350 (13.59)	169 (6.86)		
ALT (M, IQR)	30 (24–42)	21 (17–25)	16.8 (14–19)	12 (10–15)	996.773	<0.001
Normal	2,094 (83.79)	2,369 (98.42)	2,568 (99.65)	2,449 (99.43)		
Abnormal	405 (16.21)	38 (1.58)	9 (0.35)	14 (0.57)		
AST (M, IQR)	24 (19–30)	21 (18–26)	21 (18–24)	22 (19–25)	163.406	<0.001
Normal	2,330 (93.24)	2,345 (97.42)	2,542 (98.64)	2,426 (98.5)		
Abnormal	169 (6.76)	62 (2.58)	35 (1.36)	37 (1.5)		

Note

Mean (standard deviation) values and percentages are reported for continuous and categorical variables, respectively. Abnormal ALT is defined as the value of ALT >50 U/L, and Abnormal AST is the value >45 U/L. Quartiles 1, 2, 3, and 4 mean the value of AST/ALT ratio <0.9167, 0.9167–1.1578, 1.1579–1.4534, ≥1.4535, respectively. Potential means the blood pressure or blood glucose value was higher than the normal reference but not diagnosed by clinician.

### 3.2 The association of AST/ALT with chronic diseases

**FIGURE 1:**
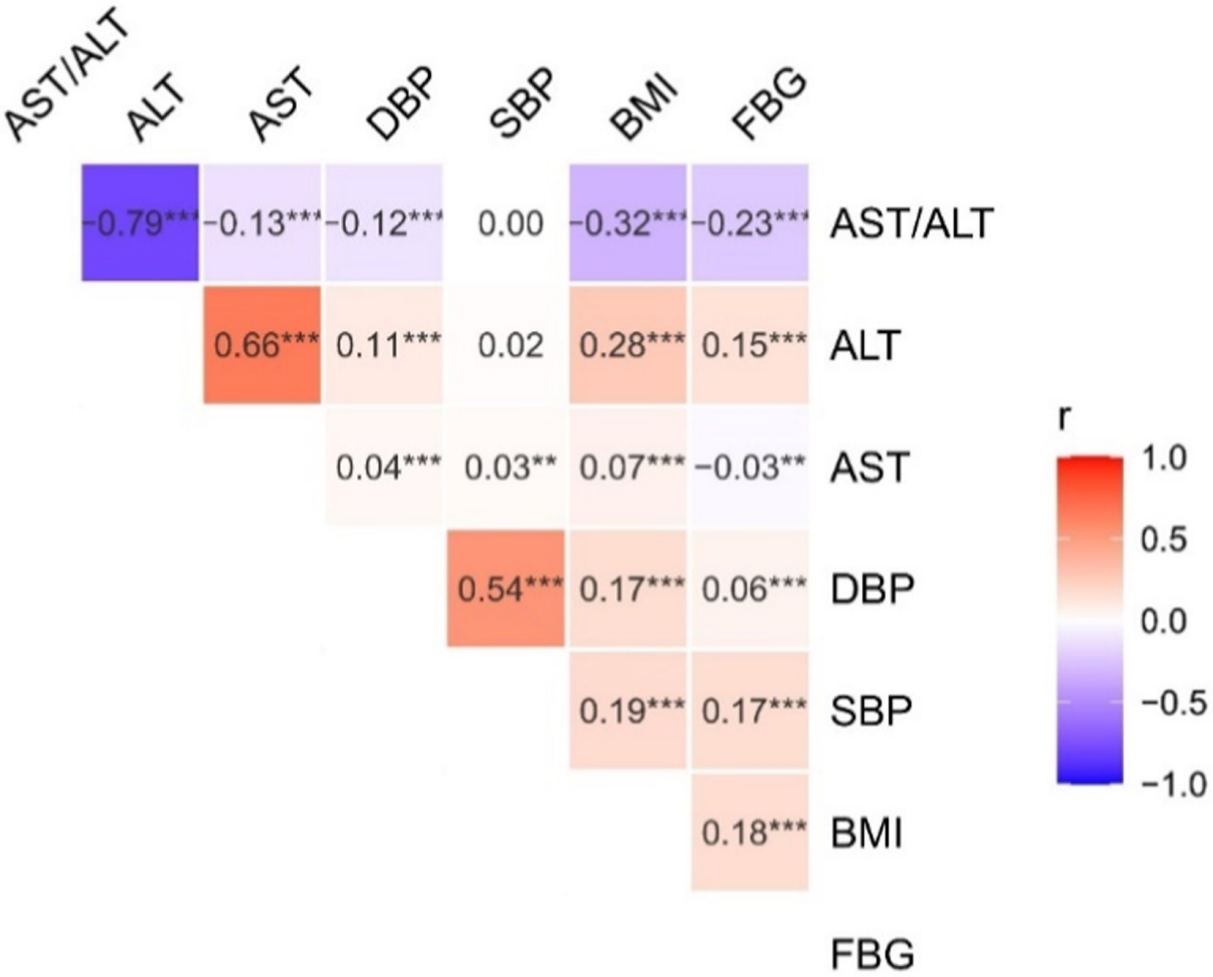
The correlation of AST/ALT ratio with the other factors. Numbers in the figure were the correlation coefficient of horizontal and vertical variables. Asterisk (***) in the figure means the correlation between two variables was statistically significant, *p *≤ 0.001. ALT, alanine transaminase; AST, aspartate aminotransferase; BMI, body mass index; DBP, diastolic blood pressure; FBG, fast blood glucose; SBP, systolic blood pressure

**FIGURE 2:**
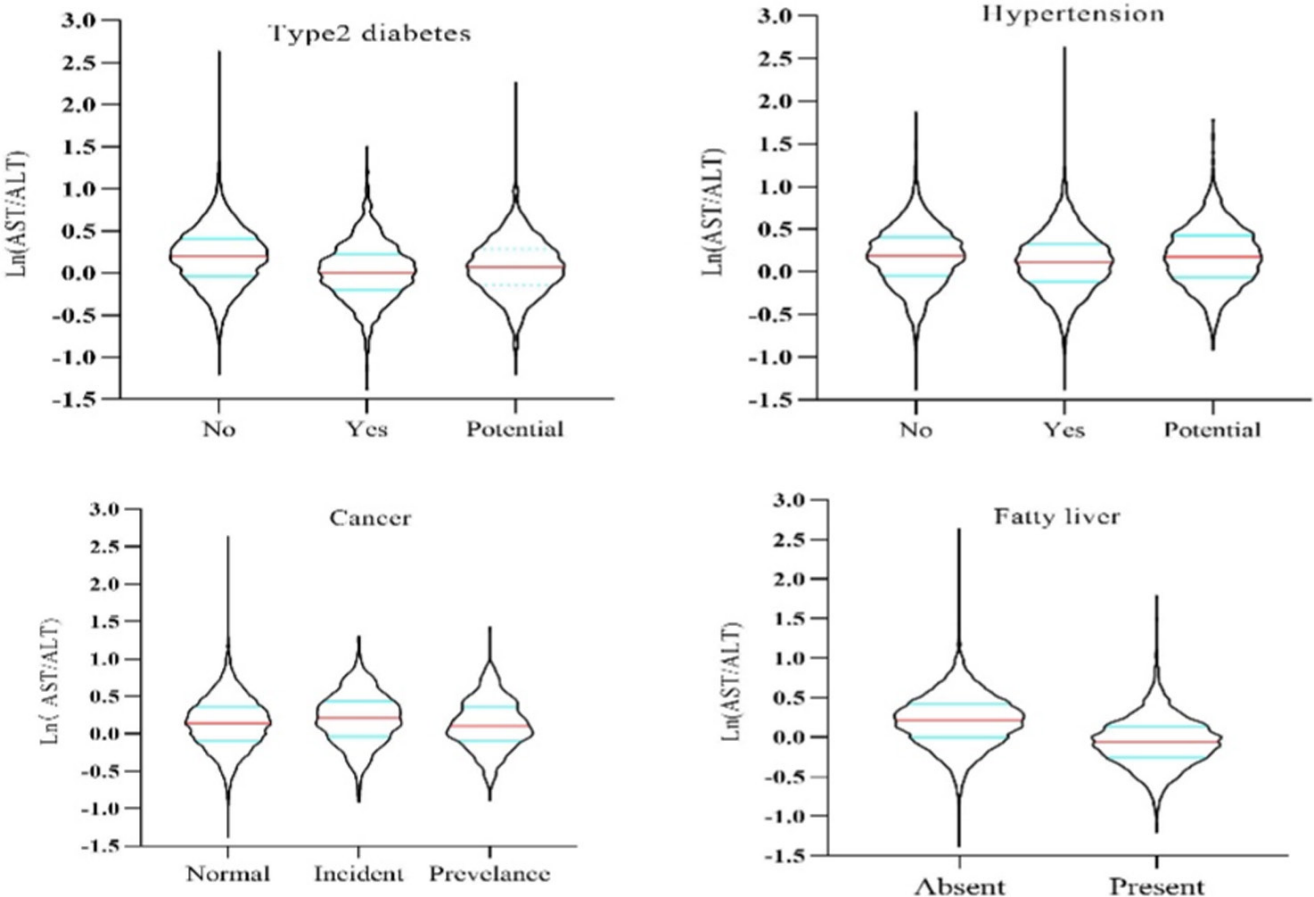
Aspartate transaminase/alanine transaminase ratio distribution in subgroups of targeted chronic diseases. The level of AST‐to‐ALT ratio was significantly decreased in patients with T2DM, hypertension, and fatty liver, but increased in patients with incident cancer

The ratio of AST to ALT was significantly higher in the normal glucose group (median: 1.22, IQR: 0.96–1.50) than in the groups with diabetes (median: 1.00, IQR: 0.82–1.25) and elevated blood glucose (median: 1.07, IQR: 0.87–1.33). According to the Spearman correlation analysis, we found that fast blood glucose (FBG) level was positively correlated with ALT (*r *= 0.15, *p *< 0.001), negatively correlated with AST (*r *= −0.03, *p *= 0.015), and significantly negatively correlated with the AST‐to‐ALT ratio (*r *= −0.23, *p *< 0.001) (Figure 1). The correlation between blood pressure and AST and ALT and their ratio showed that ALT and the AST/ALT ratio were positively associated with diastolic blood pressure (DBP). Interestingly, the baseline AST/ALT ratio of individuals developing cancers (median: 1.23, IQR: 0.96–1.54) was significantly greater than that of patients diagnosed with cancer (median: 1.11, IQR: 0.91–1.44) and individuals without cancer (median: 1.15, IQR: 0.91–1.44). As a sensitive biomarker of liver injury, the level of AST/ALT in fatty livers (median: 0.94, IQR: 0.77–1.14) was sharply lower than that in nonfatty livers (median: 1.24, IQR: 1:00–1.53). There was no difference between stroke and nonstroke individuals (Table 2, Figure 2).

**TABLE 2:** The difference in AST/ALT ratio between subjects with chronic diseases and nondiseases

Disease	No.	AST/ALT		*χ* ^2^	*p*‐Value
		Median	IQR		
Diabetes				348.44	<0.001
No	1,547	1.22	0.96–1.50		
Potential	1,629	1.07	0.87–1.33		
Yes	6,589	1	0.82–1.25		
Hypertension				90.05	<0.001
No	3,073	1.21	0.95–1.50		
Potential	1,778	1.19	0.94–1.53		
Yes	4,914	1.12	0.89–1.39		
Cancer				15.88	<0.001
No	8,947	1.15	0.91–1.44		
Prevalent	330	1.11	0.91–1.44		
Incident	488	1.23	0.96–1.54		
Fatty Liver				1111.02	<0.001
No	7,543	1.24	1.00–1.53		
Yes	2,210	0.94	0.77–1.14		
Missing	12	0.98	0.79–1.13		
Stroke				2.58	0.108
No	9,532	1.16	0.92–1.45		
Yes	221	1.21	0.93–1.50		
Missing	12	0.98	0.79–1.13		

### 3.3 A high AST/ALT ratio increases the risk of all‐cause and disease‐caused mortality

A total of 328 participants (cumulative death rate 33.59%) died based on *The Cause of Death Registration and Reporting Information System* from Taizhou Center for Disease Control and Prevention. One hundred twenty‐seven (38.72%) patients died from cancer, 97 (29.57%) from cardiac–cerebrovascular disease, 49 (14.94%) from other diseases, and 55 patients died from events including suicide, traffic accidents, and drowning.

The crude mortality rates increased with elevated AST/ALT ratios. The risk of mortality in the fourth quartile group with an AST/ALT ratio of >1.45 is 1.495 times (OR: 1.495, 95% CI: 1.003–2.228) as much as the first quartile group after adjusting covariables including age, sex, BMI, physical activity, smoking, and drinking. Older, male, and high BMI were reconfirmed as risk factors for death in the present study (Table 3). Therefore, AST/ALT ratio is an independent risk factor to predict population health risks.

**TABLE 3:** Risk of all‐cause mortality by level of AST/ALT ratio variability

	OR	95% C.I.	*p*‐Value
Age	1.652	1.230–2.218	0.001
Sex (M/F)	3.193	2.766–3.686	<0.001
BMI	1.141	1.005–1.294	0.041
Physical activity	0.877	0.796–0.967	0.008
Smoking	0.918	0.754–1.118	0.395
Drinking	0.949	0.822–1.095	0.472
AST/ALT			0.001
<0.92	1	Reference	
0.92	0.723	0.459–1.139	0.162
1.16	0.990	0.655–1.496	0.963
1.45	1.495	1.003–2.228	0.048

Note

The risk of death in fourth quartile of the AST/ALT ratio significantly increased after adjusting covariables.

### 3.4 The incidence rate of cancer was higher in the group with an increased ratio of AST/ALT

**FIGURE 3:**
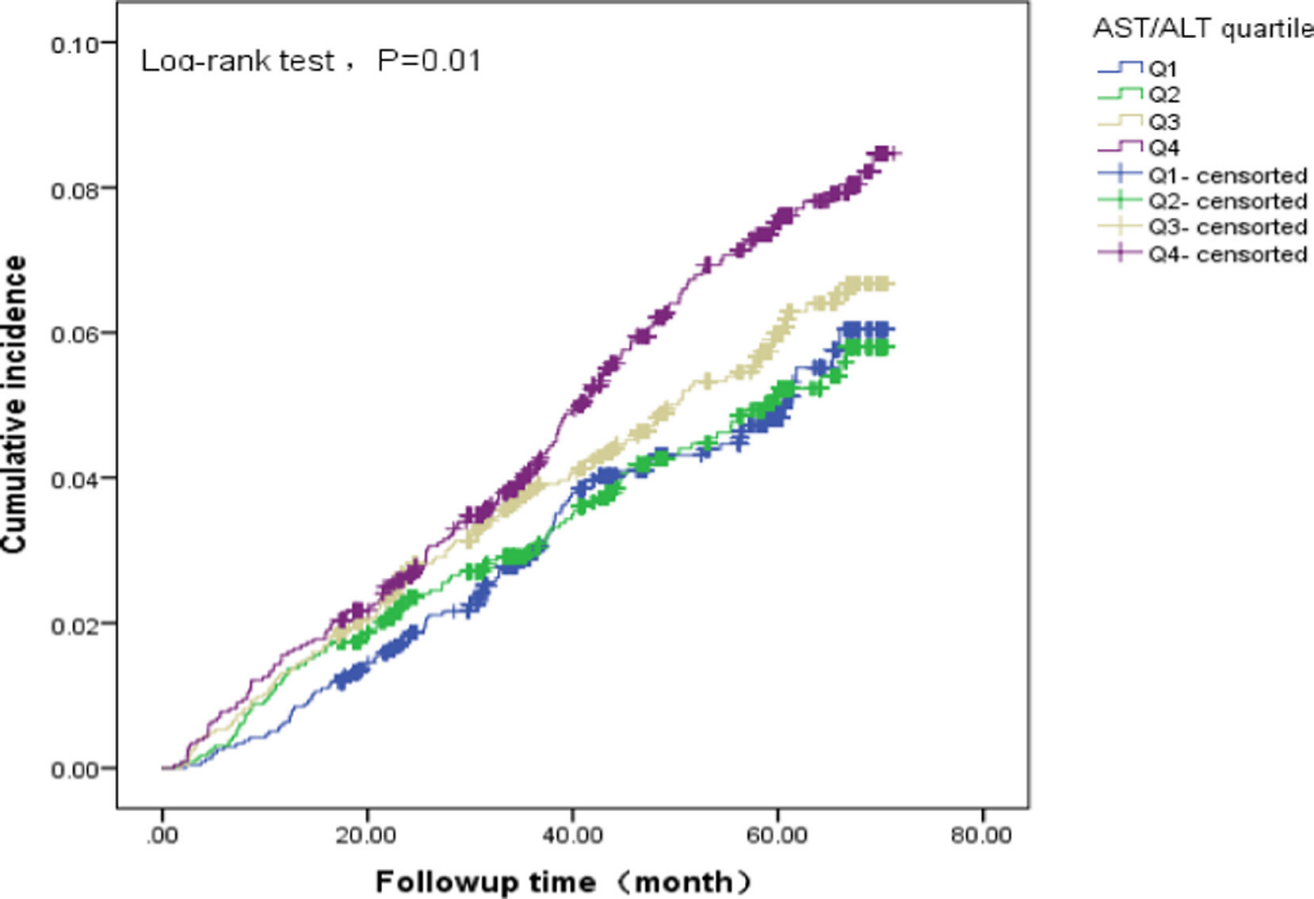
The cancer cumulative incidence among four groups of quartile AST/ALT ratio

A total of 494 patients with newly diagnosed cancer were collected in this cohort, and the cumulative incidence rate was 5.63%. The incidence rates were 4.44% for the group in the first quartile of the AST/ALT ratio, 4.93% for the second quartile, 5.62% for the third quartile, and 7.54% for the fourth quartile. The cumulative incidence rate curves were calculated by the Kaplan–Meier method, as shown in Figure 3, and there were significant differences among groups as determined by log‐rank test (*p *= 0.01).

### 3.5 Subgroup analysis on AST‐to‐ALT ratio for tumor specificity

We performed subgroup analyses according to cancer types. Among 494 incident events, 119 (24.09%) individuals developed lung cancer, 88 (17.81%) individuals developed colorectal cancer, and 42 (8.50%) individuals developed thyroid cancer, 29 (5.8%) patients developed gastric cancer, 27 (5.47%) participants developed breast cancer, 22 (4.45%) participants developed liver cancer, 21 (4.25%) participants developed prostate cancer, 13 (2.63%) participants developed pancreas cancer, 11 (2.23%) participants developed cervical cancer, and 122 (24.70%) participants developed other types of cancer. Multivariable logistic regression was employed to analyze the correlation of colorectal cancer and lung cancer risk with AST‐to‐ALT ratio. It was demonstrated in Table 4 that individuals in the fourth quartile of ratio of AST to ALT had 2.555 times (OR: 2.555, 95% CI: 1.263–5.168) risk of colorectal cancer as much as the first quartile group after adjusting covariables, but no significance was found in lung cancer, which indicated that AST‐to‐ALT ratio as a cancer risk factor has tumor specificity.

**TABLE 4:** Subgroup analysis of risk of AST‐to‐ALT ratio associated with colorectal cancer and lung cancer

Variables	Colorectal cancer			Lung cancer		
	OR	95% C.I. for OR	*p*‐Value	OR	95% C.I. for OR	*p*‐Value
Sex (M/F)	0.462	0.265–0.803	0.006	0.725	0.453–1.16	0.180
Age	1.306	1.044–1.634	0.020	1.497	1.236–1.813	<0.001
BMI	1.280	1.015–1.615	0.037	1.009	0.831–1.224	0.931
Smoking	0.863	0.641–1.162	0.331	0.828	0.627–1.093	0.182
Drinking	1.054	0.845–1.315	0.639	1.008	0.818–1.242	0.939
Physical activity	1.068	0.909–1.255	0.424	0.876	0.754–1.018	0.085
AST/ALT			0.041			0.898
<0.92	1	Reference		1	Reference	
0.92‐	1.678	0.837–3.365	0.145	0.993	0.558–1.765	0.980
1.16‐	1.384	0.667–2.871	0.383	1.169	0.673–2.032	0.579
1.45‐	2.555	1.263–5.168	0.009	1.160	0.65–2.071	0.615

## 4 DISCUSSION

In this study, we analyzed the data from routine blood test results of health exams among 9,946 community‐dwelling participants older than 40 years. We found that a high AST/ALT ratio was associated with some chronic diseases, including hypertension, T2DM, fatty liver, and cancer, but not stroke. Correlation analysis indicated that the level of ALT and the AST/ALT ratio were positively correlated with fasting blood glucose, BMI, and diastolic blood pressure. An interesting discovery was that an elevated ALT/AST ratio is a potential risk factor for cancer incidence. Furthermore, we found that the cumulative cancer incidence rate increased at a high De Ritis ratio as baseline. After linking individual data to the national all‐cause death record system, we observed significant associations between an elevated ALT/AST ratio and high mortality.

Hypertension, T2DM, fatty liver, and cancer are common chronic diseases with high prevalence in the population. Many common risk factors for these diseases, such as liver enzyme changes, can partially explain the mechanisms underlying them. The chronic diseases mentioned above are all associated with metabolic disorders.^17^ Hyperlipidemia provokes hepatocyte injury, leading to abnormal changes in serum ALT and AST levels. According to present evidence, one of the damage mechanisms is liver injury, which may impair insulin signaling, leading to a failure to suppress glucose production and ultimately hyperglycemia.^17^, ^18^, ^19^ The serum AST/ALT ratio was inversely associated with metabolic syndrome in epidemiological studies.^20^, ^21^ Our results also showed that the AST‐to‐ALT ratio was lower in the group diagnosed with T2DM or hyperglycemia than in the euglycemia group, and the hypertension population had a lower median De Ritis ratio than that of the group with normal blood pressure. Therefore, an AST‐to‐ALT ratio >1 should be considered as a clinical and preventive biomarker.

A study on the association of aminotransferase with mortality revealed that abnormal ALT or AST is associated with future mortality at the population level^22^ but did not evaluate the role of the AST/ALT ratio in the death rate. Considering the AST/ALT ratio associated with the outcome of diseases, we detected the AST/ALT ratio with disease‐specific mortality. We found that as the De Ritis ratio rose, the mortality risk also increased (OR, 1.495, 95% CI: 1.003–2.228) after adjusting for demographic and behavioral variables. Recently, several studies demonstrated the association of the AST‐to‐ALT ratio with mortality. Li et al. discovered that a higher AST/ALT ratio was an independent predictor of 1‐year mortality in polymyositis‐/dermatomyositis‐associated interstitial lung disease.^23^ Likewise, a greater AST‐to‐ALT ratio at baseline was correlated with subsequent all‐cause mortality, especially cardiovascular diseases.^21^, ^24^ Cancer survival‐related studies have shown that a high AST/ALT ratio is associated with poor outcome of renal cell carcinoma,^13^ head and neck carcinoma,^25^ and oral and oropharyngeal carcinoma^26^ among other cancers. Cardiocerebrovascular disease and cancer are the two leading causes of death in China.^27^ In terms of the association of the AST/ALT ratio with chronic diseases, it is reasonable as a predictor for mortality. The results in this study showed the individuals with high level of the AST/ALT ratio exposed high risk of mortality, but in the population with chronic diseases, the AST/ALT ratio was lower than those without chronic diseases. That result indicated the AST/ ALT ratio showed dymamic change in the different spectrum of diseases.

The present results indicated that the baseline AST/ALT ratio in the incident cancer cases was significantly higher than that in patients who were previously identified as cancer and individuals without cancer. Subsequently, cumulative incident rate analysis showed that the cancer incident rate was highest in the population in the top quartile of the AST/ALT ratio. Prior studies also reported that the patients with cancer had a higher AST/ALT ratio designed by case–control studies ^28^; our result further confirmed this result by perspective study. With the aim to detect the association of AST‐to‐ALT ratio with cancer type–specified risk, we performed subgroup analysis. The results indicated in population with higher AST‐to‐ALT ratio, the colorectal cancer risk, but not that of lung cancer, will increase. *He* and his colleague^29^ had found that higher circulating levels of ALT and AST, within a normal range, were associated with a lower risk of CRC. Our study further proved that the ratio of AST to ALT is a useful marker to estimate the risk of CRC. There was no association between the level of AST/ALT ratio and lung cancer risk, the result indicated that AST‐to‐ALT ratio has tumor type specificity. The aminotransaminases level or the ratio of AST to ALT is potential biomarker for cancer incidence. The results implied that clinicians and health promoters viewed liver function impairment as a possible signal of carcinogenesis.

Our study has several limitations. First, the years that the participants were enrolled in this study were from 2015 to 2019. However, we did not analyze the homogeneity between years due to the limited sample size. Second, observation period from 1 to 5 years is inadequate to obtain more events; longer follow‐up duration could make our results more stable. Third, the cancer incident case numbers were too small to perform subtype analysis, leading to missing information on the association of different types of cancer with the AST/ALT ratio. Totally, prospective, long‐term follow‐up studies with larger sample sizes are needed to support our results.

In conclusion, the levels of circulating liver function markers, such as ALT and AST, and their ratios in patients with chronic disease are different from those in normal patients. Higher circulating ALT/AST ratios were associated with a lower risk of mortality and cancer morbidity in this prospective study of the general population. Regardless of whether liver function markers are causal or effectual factors of disease, the AST/ALT ratio is a potential biomarker for health assessment and cancer prediction.

## CONFLICT OF INTEREST

The authors declared no conflicts of interests.

## Supporting information

Supplementary Material

## Data Availability

All data included in this study are available upon request by contact with the corresponding author. The raw data cannot be shared due to privacy or ethical restrictions.
