# Supplementary material for: Elevated AST/ALT ratio is associated with all‐cause mortality and cancer incident
Source: J Clin Lab Anal. 2022 Mar 22;36(5):e24356. doi: 10.1002/jcla.24356 (PMC9102513; doi:10.1002/jcla.24356)
Supplement: Supplementary file 1 — Supplementary Material [file JCLA-36-e24356-s001.docx]

**Supplement material**

**Sensitivity analysis**

Because of hepatobiliary diseases influence on AST and ALT level, we performed sensitivity analysis to show the distribution of AST/ALT ratio in patients with fatty liver. There were 10 cases with cholecystitis, 412 cases with gall- stone and 11 cases with liver cirrhosis, whom were excluded from sensitivity analysis. The result showed the distribution of AST/ALT ratio in patients with fatty liver was attenuated to null.

**Supplement**

**Table1 Sensitivity analysis on distribution of AST/ALT ratio in fatty liver.**

|  | **AST/ALT ratio** | | | | | | | χ^2^ | P |
| --- | --- | --- | --- | --- | --- | --- | --- | --- | --- |
|  | Quartile1(%) |  | Quartile2(%) |  | Quartile3(%) |  | Quartile4(%) |  |  |
| **Fatty liver ^a^** | | | |  |  |  |  | **1097.981** | **<0.001** |
| **No** | **1417(56.82)** |  | **1761(73.31)** |  | **2225(86.41)** |  | **2294(93.14)** |  |  |
| **Yes** | **1077(43.18)** |  | **641(26.69)** |  | **350(13.59)** |  | **169(6.86)** |  |  |
| **Fatty liver ^b^** | | | |  |  |  |  | **1076.611** | **<0.001** |
| **No** | **1355(56.91)** |  | **1683(73.56)** |  | **2135(86.97)** |  | **2220(93.32)** |  |  |
| **Yes** | **1026(43.09)** |  | **605(26.44)** |  | **320(13.03)** |  | **159(6.68)** |  |  |

Note, a means not perform sensitivity analysis; b means excluding cases with cholecystitis (10 cases), gall stone (412 cases) and liver cirrhosis(11cases).
